# Effects of different types of fluid resuscitation for hemorrhagic shock on splanchnic organ microcirculation and renal reactive oxygen species formation

**DOI:** 10.1186/s13054-015-1135-y

**Published:** 2015-12-11

**Authors:** Chun-Yu Wu, Kuang-Cheng Chan, Ya-Jung Cheng, Yu-Chang Yeh, Chiang-Ting Chien, on behalf of the NTUH Center of Microcirculation Medical Research (NCMMR)

**Affiliations:** Department of Anesthesiology, National Taiwan University Hospital, No 7, Chung-Shan S. Road, Taipei, Taiwan R.O.C; Department of Life Science, National Taiwan Normal University, No. 88, Tingzhou Road, Taipei City, Taiwan 11677 R.O.C

## Abstract

**Introduction:**

Fluid resuscitation is an indispensable procedure in the acute management of hemorrhagic shock for restoring tissue perfusion, particularly microcirculation in splanchnic organs. Resuscitation fluids include crystalloids, hypertonic saline (HTS), and synthetic colloids, and their selection affects the recovery of microcirculatory blood flow and reactive oxygen species (ROS) formation, which is often evident in the kidney, following reperfusion. In this study, the effects of acute resuscitation with 0.9 % saline (NS), 3 % HTS, 4 % succinylated gelatin (GEL), and 6 % hydroxyethyl starch (HES) 130/0.4 were compared in a hemorrhagic shock rat model to analyze restoration of microcirculation among various splanchnic organs and the gracilis muscle and reperfusion-induced renal ROS formation.

**Methods:**

A total of 96 male Wistar rats were subjected to sham operation (sham group), hemorrhagic shock (control group), and resuscitation with NS, HTS, GEL and HES. Two hours after resuscitation, changes in the mean arterial pressure (MAP), serum lactate level and the microcirculatory blood flow among various splanchnic organs, namely the liver, kidney, and intestine (mucosa, serosal muscular layer, and Peyer’s patch), and the gracilis muscle, were compared using laser speckle contrast imaging. Renal ROS formation after reperfusion was investigated using an enhanced in vivo chemiluminescence (CL) method.

**Results:**

Microcirculatory blood flow was less severely affected by hemorrhaging in the liver and gracilis muscle. Impairment of microcirculation in the kidney was restored in all resuscitation groups. Resuscitation in the NS group failed to restore intestinal microcirculation. Resuscitation in the HTS, GEL, and HES groups restored intestinal microcirculatory blood flow. By comparison, fluid resuscitation restored hemorrhagic shock-induced hypotension and decreased lactatemia in all resuscitation groups. Reperfusion-induced in vivo renal ROS formation was significantly higher in the GEL and HES groups than in the other groups.

**Conclusion:**

Although fluid resuscitation with NS restored the MAP and decreased lactatemia following hemorrhagic shock, intestinal microcirculation was restored only by other volume expanders, namely 3 % HTS, GEL, and HES. However, reperfusion-induced renal ROS formation was significantly higher when synthetic colloids were used.

**Electronic supplementary material:**

The online version of this article (doi:10.1186/s13054-015-1135-y) contains supplementary material, which is available to authorized users.

## Introduction

Fluid resuscitation is often the *sine qua non* treatment in the acute management of hemorrhagic shock. However, even when a sufficient amount of fluid is administered for restoring hemodynamic stability, splanchnic organ injury may persist. This may be because different types of resuscitation fluid may differently affect the recovery of microcirculatory blood flow and reperfusion-induced reactive oxygen species (ROS) formation [1, 2]. During resuscitation, adequate organ perfusion is more strongly correlated with microcirculatory improvement than macrocirculatory improvement [3]. Accordingly, numerous clinical investigations have been conducted to clarify the microcirculatory effects of different types of resuscitation fluid, including crystalloids, hypertonic saline (HTS), and synthetic colloids, by observing sublingual microcirculation [1, 4]. However, because splanchnic microcirculation is partly compromised during hypovolemia, which may participate in the development of multiple-organ dysfunction syndrome [5], and the splanchnic microcirculatory response to fluid challenge may become dissociated from the sublingual microcirculatory response [6, 7], the effects of different types of fluid on the splanchnic microcirculation during resuscitation from hemorrhagic shock remain unclear. Accordingly, we previously used an experimental model to investigate the microcirculation among multiple splanchnic organs during hemorrhagic shock and 0.9 % saline resuscitation and observed that the intestinal microcirculation remained impaired despite the recovery of the macrocirculation [8]. Thus far, the microcirculatory effects of other volume expanders, such as HTS and synthetic colloids, among multiple splanchnic organs remain unexplored.

In addition to microcirculatory change, reperfusion after fluid resuscitation is another factor influencing organ injury. The kidney is one of the most sensitive splanchnic organs targeted in reperfusion-mediated oxidative tissue injury [9]. ROS formation is an early biomarker of reperfusion-induced oxidative stress and may be detectable in the acute phase of fluid resuscitation. Excess ROS formation is associated with systemic inflammation and can initiate cell death [2]; moreover, it is closely correlated to renal injury [9, 10] The extent of ROS formation after reperfusion may depend on the type of resuscitation fluid used [2], and fluid resuscitation using synthetic colloids is relevant to acute kidney injury [11–13]. However, renal ROS formation during the acute phase of fluid resuscitation using synthetic colloids is less thoroughly investigated compared with that using other types of resuscitation fluid.

Therefore, in the current study, we used different types of resuscitation fluids, namely 0.9 % saline, 3 % HTS, 4 % succinylated gelatin (GEL), and 6 % hydroxyethyl starch (HES) 130/0.4, for acute resuscitation in a hemorrhagic shock rat model. The primary goal of this study was to determine the effects of different resuscitation fluids on the restoration of microcirculation in multiple splanchnic organs, using the laser spackle contrast imaging (LSCI) technique. The secondary goal was to calculate renal reperfusion injury-induced ROS formation by using an in vivo ROS assessment technique.

## Methods

### Experimental animals

A total of 96 male Wistar rats (body weight = 250 ± 30 g; Biolasco Taiwan Co., Taipei, Taiwan) were used. The rats were kept on a 12-h light–dark cycle and had a free access to water and food. All experimental procedures were approved by the Institutional Animal Care and Use Committee of National Taiwan University and were conducted in accordance with its guidelines.

### Part I - changes of microcirculatory blood flow intensity in splanchnic organs

#### Anesthesia and surgical procedure

The rats were anesthetized and prepared as described in our previous study [8]. A tracheostomy was performed, and a 14G catheter (Surflo; Terumo Corporation, Laguna, Philippines) was inserted into the trachea to maintain spontaneous breathing. Anesthesia was maintained using 1.2 % inhaled isoflurane. Subcutaneous atropine (0.05 mg/kg in 10 mL/kg of saline) was injected to limit the rate of moisture evaporation from the surgical open wound and to prevent airway secretion. The body temperature of the rats was continuously monitored rectally, and a warmer pad was applied to maintain it between 36 and 37 °C. Polyethylene catheters (PE-50; Intramedic 7411, Clay Adams, Parsippany, NJ, USA) were inserted into the right common carotid artery and external jugular vein. The catheter in the right common carotid artery was used to continuously monitor macrocirculatory hemodynamics, including the mean arterial pressure (MAP) and heart rate (HR), and to withdraw blood for inducing hemorrhagic shock. The external jugular vein was used to infuse resuscitation fluid.

A long, midline laparotomy was performed to exteriorize splanchnic organs, including the liver, left kidney, and a segment of the terminal ileum (approximately 6–10 cm in length, proximal to the ileocecal valve). A 2-cm section was made on the antimesenteric aspect of the intestinal lumen by using a high-frequency desiccator (Aaron 900; Bovie Aaron Medical, St. Petersburg, FL, USA) for carefully exposing the opposing mucosa to study microcirculation. Furthermore, the nearby intestinal serosal muscular layer (at the midline of the antimesenteric aspect) and the central Peyer’s patch (identified by visualizing the lymph nodes) were identified to study microcirculation. Moreover, the right gracilis muscle was exposed for analyzing microcirculatory changes relative to the splanchnic organs. The exposed viscera and tissue were kept moist by treating them every hour with 0.5 mL of aerosolized saline, prewarmed to 37 °C.

#### Evaluation of microcirculatory blood flow

The microcirculation was assessed using the LSCI technique [14] and the setup was similar to that employed in our previous study [8]. A full-field laser perfusion imager (MoorFLPI; Moor Instruments, Ltd., Devon, UK) was used to continuously record the intensity of microcirculatory blood flow in the splanchnic organs, starting from the baseline. The imager uses LSCI, in which a random speckle pattern is generated when a tissue is illuminated by a laser light. The random speckle pattern changes when blood cells move within the regions of interest (ROIs). When the level of movement is high (high flow), the changing pattern becomes more blurred, and the contrast in that particular region declines accordingly. Therefore, low contrast is associated with high flow, and high contrast is associated with low flow. The contrast image is processed to produce a 16-color-coded image that is associated with the blood flow in the tissue (e.g., blue indicates low flow and red, high flow). The microcirculatory blood flow intensity of each ROI was recorded as a perfusion unit (PU), which is equal to the product of the average speed and concentration of red blood cells moving in the tissue sample volume (i.e., blood cell flux or perfusion). The images were recorded and analyzed in real time using the MoorFLPI Version 3.0 software (Moor Instruments, Ltd.). Six separate ROIs were defined on the liver, left kidney, intestinal mucosa, serosal muscular layer, Peyer’s patch, and right gracilis muscle (details on the selection of ROIs are listed in Additional file 1). The intensity of microcirculatory blood flow was recorded as an arbitrary PU.

### Hemorrhagic shock and fluid resuscitation protocols

In total, 60 rats were randomly assigned to the following six groups (n = 10 in each group): (1) the sham group, in which the rats received all surgical procedures except blood withdrawal and fluid resuscitation; (2) the control group, in which the rats underwent hemorrhaging but no fluid resuscitation; (3) the NS group, in which the rats were resuscitated with 0.9 % saline; (4) the HTS group, in which the rats were resuscitated with 3 % HTS; (5) the GEL group, in which the rats were resuscitated with 4 % succinylated GEL (Gelofusine®, B. Braun, Taiwan); and (6) the HES group, in which the rats were resuscitated with 6 % HES 130/0.4 (Voluven®, Fresenius-Kabi, Bad Homberg, Germany).

**Fig. 1:**
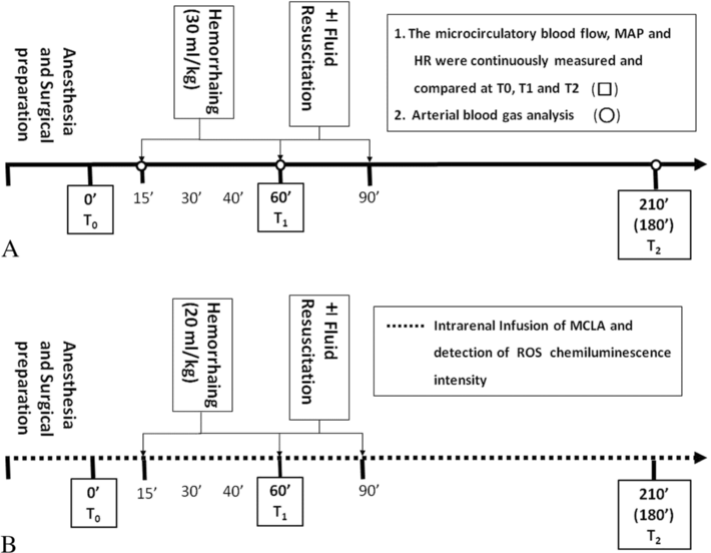
Timeline of the protocols for part I of the experiment for assessing splanchnic organ microcirculation (**a**) and part II of the experiment for calculating renal reactive oxygen species (*ROS*) formation after reperfusion (**b**). *T*_*0*_ baseline state, *T*_*1*_ complete of hemorrhagic shock, *T*_*2*_ end of measurement (2 h after shock (180’ minutes) or 2 h after resuscitation (210’ minutes)), *MAP* mean arterial pressure, *HR* heart rate

Figure 1a shows a timeline of part I of the experiment. After completion of surgery, the rats were allowed to stabilize for 30 minutes before the baseline measurements were recorded (baseline condition was considered stable when all measurement values remained at 10 % for 15 minutes; defined as 0’ minutes; T_0_). At T_0_, the concentration of isoflurane was lowered to 0.7 % to prevent over-anesthesia in the rats without further surgical stimulation, and hemorrhagic shock was then induced through controlled blood withdrawal via a right carotid arterial cannula with total blood loss of 30 mL/kg from 15’ to 60’ (T_1_) minutes. Fluid resuscitation was administrated from 60’ to 90’ minutes, and the volume of fluid used for resuscitation was same as that of blood withdrawn. Two hours after fluid resuscitation, part I of the experiment was complete (T_2_; at 210’ minutes in the rats receiving fluid resuscitation and at 180’ min in the rats in the sham and control groups; Fig. 1a). Hemodynamic measurements of the macrocirculation and microcirculation were compared among the groups at T_0_, T_1_, and T_2_ (Fig. 1a). Because different organs may have different baseline values of the microcirculatory blood flow intensity, the percent changes in the microcirculatory blood flow intensity at T_1_ and T_2_ were compared with the T_0_ baseline values.

The first 0.2 mL of blood withdrawn at 15’ minutes and the final 0.2 mL of blood withdrawn at T_1_ were analyzed using arterial blood gas analysis as the baseline status and shock status, respectively. At the end of the measurement (T_2_), arterial blood gas analysis was repeated to evaluate the effects of fluid resuscitation.

### Part II *-* formation of renal reactive oxygen species in vivo

For investigating kidney ROS activity in vivo, 36 rats underwent the same hemorrhagic shock and fluid resuscitation procedures (n = 6 rats in each group) as did the rats used for investigating microcirculatory changes in the splanchnic organs with two changes. First, the rats were anesthetized with a subcutaneous urethane injection (1.2 g/kg) instead of volatile anesthesia because in vivo measurement was conducted in a closed box that did not allow the entry of a volatile anesthesia breathing circuit. In addition, volatile anesthesia may mask ROS formation because of its antioxidant properties [15]. Second, pilot experiments showed that rats receiving anesthesia with subcutaneous urethane could survive only if the volume of blood loss was lowered. Therefore, hemorrhagic shock was induced by withdrawing a lesser amount of blood (20 mL/kg; Fig. 1b).

The method for detecting chemiluminescence (CL) from the organ surface after intrarenal arterial infusion of a superoxide anion probe, 2-methyl-6-[4-methoxyphenyl]-3,7-dihydroimidazo-[1,2-a]-pyrazin-3-one hydrochloride (MCLA) (TCI-Ace; Tokyo Kasei Kogyo Co. Ltd., Tokyo, Japan) [16, 17], was adapted to demonstrate ROS production in kidneys subjected to ischemia reperfusion. For excluding photon emission from sources other than the kidney, the rats were housed in a dark box with a shielded plate. Only the renal window was left unshielded and was positioned under a reflector, which reflected the photons from the exposed kidney surface onto the detector area. A single dose of N,N9-dimethyldiacridium (1 mM in 0.1 mL) (lucigenin; Sigma) or a continuous infusion of MCLA (0.2 mg/mL per h) was administered through an intrarenal arterial catheter. The lucigenin- or MCLA-enhanced CL signal from the kidney surface was measured continuously during administration using a CL analyzer (CLD-110, Tohoku Electronic Industrial Co., Sendai, Japan). The ROS response was directly evaluated from the kidney surface through intrarenal arterial infusion of MCLA (0.2 mg/mL/h) [16]. At T_2_, the increased ROS formation from baseline was compared.

### Statistical analysis

Analysis was performed using statistical software (MedCalc Inc., Mariakerke, Belgium) and SigmaPlot for Windows, Version 12 (SAS Institute, Cary, NC, USA). Data are presented as the median (interquartile range) in tables and the mean (standard error of the mean) in figures. According to our previous report [8], the sample size was estimated to be six rats in each group to detect a difference in the mean microcirculatory blood flow of 200 PU. Mean differences and percent differences at different time points among the six groups were investigated using repeated measurement analysis of variance (ANOVA) with the factors of time and group, followed by the Bonferroni test. A *p* value <0.05 was considered statistically significant.

## Results

### Part I - changes in microcirculatory blood flow intensity in splanchnic organs

#### Microcirculatory blood flow changes

**Fig 2:**
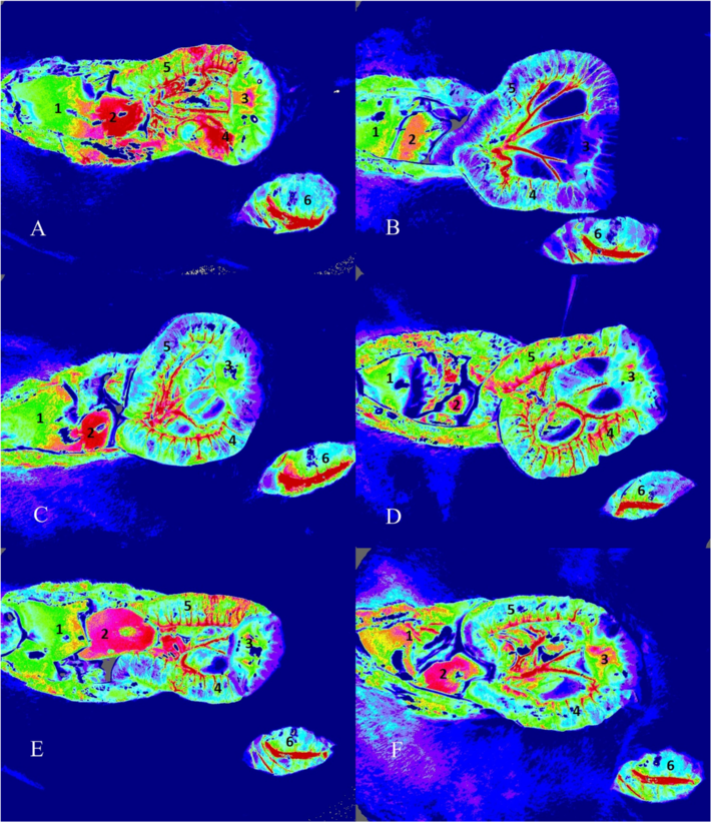
Example of laser speckle contrast imaging of the microcirculatory blood flow intensity in the sham (**a**), control (**b**), 0.9 % saline (**c**), hypertonic saline (**d**), gelatin (**e**), and hydroxyethyl starch (**f**) groups at time (T)_2_. The regions of interest were the liver (1), kidney (2), intestinal mucosa (3), serosal muscular layer (4), Peyer’s patch (5), and gracilis muscle (6)

**Fig 3:**
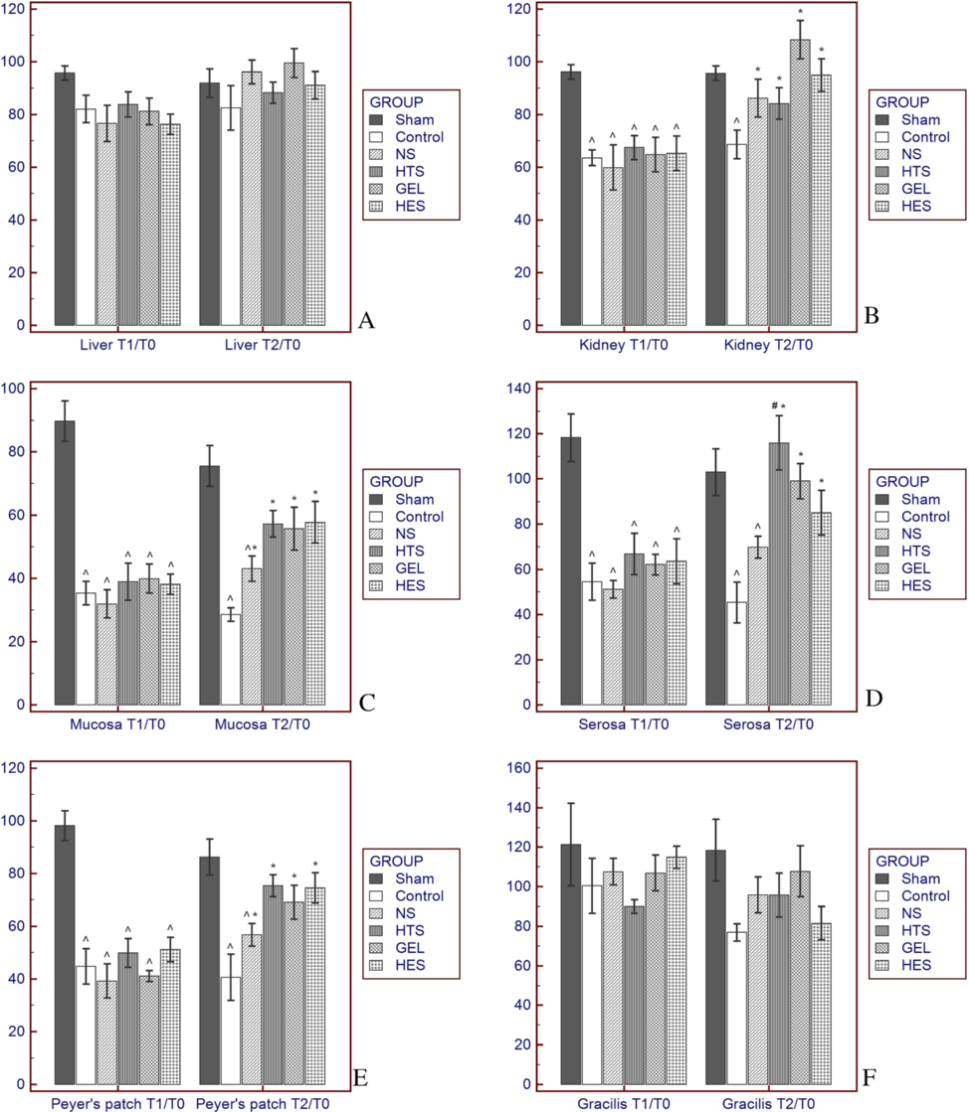
Percent changes in the microcirculatory blood flow intensity at time (T)_1_ and T_2_ compared with T_0_ in the liver (**a**), kidney (**b**), intestinal mucosa (**c**), serosal muscular layer (**d**), Peyer’s patch (**e**) and gracilis muscle (**f**). *Value in a fluid resuscitation group significantly different from that in the control group with *p* <0.05. ^#^Value in a fluid resuscitation group significantly different from that in the 0.9 % saline group with *p* <0.05. ^^^Value in a fluid resuscitation group significantly different from that in the sham group with *p* <0.05. *NS* 0.9 % saline, *HTS* hypertonic saline, *HES* hydroxyethyl starch, *GEL* gelatin

Table 1 shows a summary of changes in the sequential absolute values of the intensity of microcirculatory blood flow during the measurement period. The rats in the sham group had no significant changes in the intensity of microcirculatory blood flow in all investigated organs (Table 1; Figs. 2a and 3a–f). The microcirculatory blood flow in the liver and gracilis muscle did not significantly change after hemorrhagic shock and fluid resuscitation (Table 1; Fig. 3a, f). Hemorrhagic shock significantly lowered the intensity of microcirculatory blood flow in the kidney and intestine (including the mucosa, serosal muscular layer, and Peyer’s patch; Figs. 2b and 3b–e).

**Table 1:** Microcirculatory blood flow intensity changes after hemorrhagic shock and fluid resuscitation in part I of the experiment

Flux	T_0_	T_1_	T_2_	ANOVA group effects	ANOVA time effects	ANOVA group-time interaction
	Baseline	Completion of shock	End of experiment			
Liver				*p* = 0.375	*p* <0.001	*p* = 0.132
Sham	1176 (1064–1245)	1147 (1091–1233)	1048 (938–1210)			
Control	1115 (1052–1316)	856 (796–1203)	1082 (849–1095)			
NS	1314 (1148–1365)	1026 (854–1116)	1174 (1136–1304)			
HTS	1230 (1177–1317)	1027 (897–1200)	1019 (938–1239)			
GEL	1095 (1063–1166)	922 (828–1033)	1111 (1027–1185)			
HES	1250 (1044–1339)	898 (826–960)	1181 (1029–1228)			
Kidney				*p* = 0.012	*p* <0.001	*p* <0.001
Sham	1653 (1443–1708)	1596 (1490–1712)	1479 (1458–1539)			
Control	1476 (1260–1619)	894 (774–1104)^***^	1041 (673–1295)^***^			
NS	1604 (1581–1821)	1071 (805–1228)^***^	1503 (1296–1617)^*^			
HTS	1603 (1570–1695)	1116 (974–1283)^***^	1413 (1171–1645)^*^			
GEL	1601 (1429–1642)	1093 (860–1211)^***^	1687 (1448–1862)^*^			
HES	1537 (1132–1709)	1062 (857–1253)^***^	1478 (1195–1596)^*^			
Mucosa				*p* < 0.001	*p* < 0.001	*p* < 0.001
Sham	1422 (1238–1700)	1259 (1189–1332)	1150 (960–1187)			
Control	144 (1244–1749)	441 (404–668)^***^	398 (366–434)^***^			
NS	1913 (1737–2112)	631 (387–831)^***^	763 (728–872)^*, ***^			
HTS	1732 (1552–1978)	599 (507–895)^***^	950 (858–1123)^*^			
GEL	2015 (1723–2130)	768 (620–802)^***^	980 (867–1061)^*^			
HES	1682 (1393–1872)	567 (520–817)^***^	950 (818–1247)^*^			
Serosa				*p* = 0.010	*p* <0.001	*p* <0.001
Sham	933 (903–986)	1133 (920–1215)	937 (675–1306)			
Control	950 (803–1003)	446 (349–681)^***^	369 (267–704)^***^			
NS	1049 (901–1090)	563 (441–646)^***^	669 (653–759)^***^			
HTS	1002 (840–1080)	612 (460–692)^***^	1063 (809–1456)^*, **^			
GEL	887 (863–1016)	568 (477–691)^***^	804 (725–1058)^*^			
HES	854 (850–1031)	526 (426–659)^***^	759 (628–1017)^*^			
Peyer’s patch				*p =* 0.002	*p* <0.001	*p* <0.001
Sham	1513 (1220–1941)	1511 (1220–1941)	1328 (1146–1563)			
Control	1149 (1052–1318)	616 (421–636)^***^	548 (436–668)^***^			
NS	1530 (1261–1740)	581 (348–907)^***^	916 (718–1040)^*, ***^			
HTS	1603 (1549–1739)	838 (655–999)^***^	1223 (1046–1441)^*^			
GEL	1578 (1309–1725)	655 (607–875)^***^	1075 (919–1080)^*^			
HES	1525 (1443–1669)	791 (634–934)^***^	1209 (837–1513)^*^			
Gracilis muscle				*p =* 0.108	*p =* 0.018	*p* <0.001
Sham	941 (928–999)	989 (868–1371)	1091 (857–1367)			
Control	897 (856–1087)	841 (641–1066)	721 (634–797)			
NS	991 (904–1190)	967 (791–1268)	975 (869–1140)			
HTS	1020 (932–1175)	950 (766–1025)	967 (866–1131)			
GEL	1009 (884–1077)	1008 (853–1147)	953 (877–1252)			
HES	1064 (904–1223)	1302 (1152–1397)	798 (695–1044)			

Data are median (interquartile range). *Value in the fluid resuscitation group significantly different to control group with *p* <0.05. **Value in fluid resuscitation group significantly different to 0.9 % saline group with *p* <0.05. ***Value in fluid resuscitation or control significantly different to sham group with *p* <0.05. *ANOVA* analysis of variance, *T* time, *NS* 0.9 % saline, *HTS* hypertonic saline, *HES* hydroxyethyl starch, *GEL* gelatin

The intensity of microcirculatory blood flow in the kidney was restored after fluid resuscitation in the NS, HTS, GEL, and HES groups at T_2_ (Figs. 2c–f and 3b). Fluid resuscitation by NS induced significantly improved intestinal mucosal and Peyer’s patch microcirculatory blood flow compared with that in the control group (Fig. 3c and e). However, the microcirculation of the intestine (including the mucosa, serosal muscular layer, and Peyer’s patch) in the NS group remained significantly impaired compared with that in the sham group at T_2_ (Figs. 2c and 3c–e). By comparison, the intensity of microcirculatory blood flow in the intestine in the HTS (Figs. 2d and 3c–e), GEL (Figs. 2e and 3c–e), and HES (Figs. 2f and 3c–e) groups significantly improved relative to that of the control group and was comparable with that of the sham group at T_2_. The difference in microcirculatory blood flow in the serosal muscular layer between the HTS and the NS groups at T_2_ was also statistically significant (Fig. 3d).

#### Macrocirculation and arterial blood gas analysis

The macrocirculatory changes after hemorrhagic shock and fluid resuscitation in part I of the experiment are summarized in Table 2. The repeated measurement ANOVA showed that there were significant differences in MAP change between the control and other groups (Table 2; *p* <0.001). The hemorrhagic shock induced a significant reduction in the MAP from 115 (100–118) mmHg at T_0_ to 56 (46–67) mmHg at T_2_ in the control group (*p* <0.001). Fluid resuscitation restored the MAP to 80 (76–85) mmHg, 89 (70–102) mmHg, 89 (82–93) mmHg and 85 (72–99) mmHg at T_2_ in the NS, HTS, GEL and HES groups, respectively (compared to the control group, each *p* <005; Table 1). Significant changes in heart rate were not found between groups.

**Table 2:** Macrocirculatory changes after hemorrhagic shock and fluid resuscitation in part I of the experiment

	T_0_	T_1_	T_2_	ANOVA group effects	ANOVA time effects	ANOVA group-time interaction
	Baseline	Completion of shock	End of experiment			
MAP (mmHg)				*p* <0.001	*p* <0.001	*p* <0.001
Sham	104 (101–109)	109 (101–112)^*^	103 (80–107)			
Control	115 (100–118)	61 (46–74)	56 (46–67)^**^			
NS	103 (96–112)	61 (57–69)^**^	80 (76–85)			
HTS	104 (97–112)	68 (60–75)^**^	89 (70–102)^*^			
GEL	108 (100–120)	66 (54–85)^**^	89 (82–93)^*^			
HES	96 (90–105)	59 (55–64)^**^	85 (72–99)^*^			
HR (/min)				*p =* 0.394	*p* <0.001	*p =* 0.153
Sham	361 (357–379)	375 (333–422)	355 (340–384)			
Control	389 (356–400)	365 (342–379)	410 (358–416)			
NS	346 (326–357)	341 (287–361)	346 (337–392)			
HTS	365 (342–385)	348 (312–362)	400 (371–412)			
GEL	379 (352–400)	340 (291–344)	370 (333–379)			
HES	352 (346–376)	344 (323–384)	348 (332–382)			

Data are median (interquartile range). *Value significantly higher than in the control group with *p* <0.05. **Value in fluid resuscitation or control group significantly different to sham group with *p* <0.05. *ANOVA* analysis of variance, *T* time, *MAP* mean arterial pressure, *HR* heart rate, *NS* 0.9 % saline, *HTS* hypertonic saline, *HES* hydroxyethyl starch, *GEL* gelatin

Hemorrhagic shock induced significant decreases in arterial pH value at T_1_ in each group. The respiratory parameters including the arterial partial pressure of oxygen (PaO_2_)/inspired fraction of oxygen (FiO_2_) ratio and arterial partial pressure of carbon dioxide (PaCO_2_) were comparable between each group. Hemorrhagic shock induced anemia in each group such that the hemoglobin level and hematocrit decrease from 13.0 (12.5–13.8) g/dL and 39 (38–41) % at T_0_ to 10.0 (8.6–10.2) g/dL and 30 (26–30) % at T_2_ in the control group. The absolute hemoglobin and hematocrit values at T_2_ were higher in the GEL group than those in the other groups, but the changes in values from T_0_ to T_2_ were comparable between each group (Table 3). Hemorrhagic shock induced lactatemia in each group such that the serum lactate level increased from 0.6 (0.5–0.9) mmol/L at T_0_ to 4.3 (3.0–5.8) mmol/L at T_2_ in the control group. (Table 3). The serum lactatemia at T_2_ was comparably decreased in all resuscitation groups (Table 3).

**Table 3:** Arterial blood gas analysis in part I of the experiment

	T_0_	T_1_	T_2_	ANOVA group effects	ANOVA time effects	ANOVA group-time interaction
	Baseline	Completion of shock	End of experiment			
pH				*p =* 0.520	*p =* 0.032	*p =* 0.527
Control	7.38 (7.36-7.40)	7.36 (7.31-7.38)	7.37 (7.34-7.44)			
NS	7.40 (7.39-7.42)	7.38 (7.34-7.39)	7.42 (7.40-7.44)			
HTS	7.38 (7.37-7.40)	7.36 (7.35-7.38)	7.39 (7.31-7.39)			
GEL	7.38 (7.35-7.40)	7.34 (7.30-7.36)	7.36 (7.32-7.39)			
HES	7.39 (7.38-7.41)	7.35 (7.31-7.37)	7.40 (7.37-7.42)			
PaO_2_/FiO_2_				*p =* 0.182	*p =* 0.168	*p =* 0.738
Control	481 (439–499)	390 (378–444)	430 (350–458)			
NS	421 (384–468)	425 (391–470)	442 (376–467)			
HTS	472 (454–490)	464 (410–486)	485 (450–533)			
GEL	434 (431–456)	421 (385–458)	426 (380–510)			
HES	412 (399–466)	399 (344–467)	413 (386–478)			
PaCO_2_ (mmHg)				*p =* 0.491	*p =* 0.484	*p =* 0.351
Control	38.4 (37.4-40.4)	36.1 (34.7-44.9)	34.2 (27.9-34.5)			
NS	37.7 (36.7-39.6)	36.3 (33.8-41.9)	34.3 (31.2-37.7)			
HTS	38.7 (37.2-40.4)	38.1 (35.6-40.6)	36.8 (35.5-39.5)			
GEL	38.0 (32.3-39.3)	37.8 (34.8-39.4)	39.0 (35.6-43.1)			
HES	39.7 (39.4-41.1)	34.4 (30.9-44.8)	37.3 (37.0-47.0)			
Hemoglobin (g/dL)				*p =* 0.021	*p* < 0.001	*p =* 0.098
Control	13.0 (12.5-13.8)	9.5 (9.4-10.0)	10.0 (8.6-10.2)			
NS	12.6 (12.4-12.9)	10.1 (7.2-10.9)	9.1 (8.5-9.4)			
HTS	12.4 (11.8-12.6)	9.8 (9.2-10.4)	8.5 (8.4-9.0)			
GEL	12.6 (12.3-13.4)	10.0 (9.9-10.6)	11.2 (8.8-12.4)^*^			
HES	12.4 (12.0-12.8)	10.1 (9.6-10.9)	8.8 (8.3-9.3)			
Hematocrit (%)				*p =* 0.021	*p* <0.001	*p =* 0.120
Control	39 (38–41)	29 (28–30)	30 (26–30)			
NS	38 (37–39)	30 (22–33)	27 (25–29)			
HTS	37 (35–38)	29 (27–32)	26 (25–27)			
GEL	38 (37–38)	30 (30–32)	34 (27–37)			
HES	38 (36–39))	30 (29–33)	27 (25–28)			
Lactate (mmol/L)				*p =* 0.026	*p* <0.001	*p* <0.001
Control	0.6 (0.5-0.9)	4.4 (3.8-5.0)	4.3 (3.0-5.8)			
NS	1.0 (1.0-1.3)	4.3 (4.0-5.0)	2.0 (1.8-2.6)^*^			
HTS	0.9 (0.8-1.4)	4.6 (3.0-5.6)	1.7 (1.6-2.2)^*^			
GEL	1.2 (0.9-1.5)	3.5 (2.3-4.5)	1.2 (1.0-1.7)^*^			
HES	1.3 (1.0-1.3)	3.3 (3.1-4.1)	1.9 (1.3-2.4)^*^			

Data are median (interquartile range). *Value significantly different to the control group with *p* <0.05. *ANOVA* analysis of variance, *T* time, *NS* 0.9 % saline, *HTS* hypertonic saline, *HES* hydroxyethyl starch, *GEL* gelatin, *PaO*_*2*_ */FiO*_*2*_ ratio of arterial partial pressure of oxygen/inspired oxygen fraction, *PaCO*_*2*_ arterial partial pressure of carbon dioxide

### Part II formation of renal reactive oxygen species in vivo

**Fig 4:**
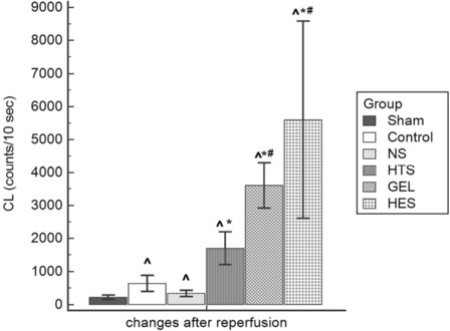
Comparison of the amount of reperfusion-induced formation of renal reactive oxygen species in vivo after fluid resuscitation. *Value in a fluid resuscitation group significantly different from that in the control group with *p* <0.05. ^#^Value in a fluid resuscitation group significantly different from that in the other fluid resuscitation group, including the 0.9 % saline (*NS*) and hypertonic saline (*HTS*) groups with *p* <0.05. ^^^Value in a fluid resuscitation group significantly different from that in the sham group with *p* <0.05. *CL* chemiluminescence, GEL gelatin, *HES* hydroxyethyl starch

Hemorrhagic shock induced a significant increase in renal ROS formation in vivo (551 (322–955) vs. 155 (155–303) CL counts/10 s in the control and the sham groups respectively; *p* <0.05). Fluid resuscitation also significantly increased in vivo renal ROS formation in the NS, HTS, GEL, and HES groups compared with the sham group at T_2_ (277 (189–480), 1644 (1065–2344), 3918 (2596–4610), and 3119 (1880–9298) CL counts/10 s, in the NS, HTS, GEL, and HES groups, respectively; *p* <0.05 compared with the sham group; Fig. 4). Reperfusion-induced renal ROS formation was higher in the HTS, GEL, and HES groups than in the control group. Specifically, the GEL and the HES groups had significantly higher in vivo renal ROS formation compared with that in the HTS and other groups at T_2_ (Fig. 4). The amount of renal ROS formation was comparable between the GEL and HES groups at T_2_.

## Discussion

In the current study, we compared commonly used resuscitation fluids, namely a crystalloid (0.9 % saline), HTS, and synthetic colloids (GEL and HES), in the acute management of hemorrhagic shock. The major findings are that first, we observed that although fluid resuscitation with the crystalloid restored the MAP and decreased the serum lactatemia, intestinal microcirculation was effectively resuscitated only after using the HTS or synthetic colloids; second, fluid resuscitation using the synthetic colloids was associated with the greatest formation of renal ROS in vivo after reperfusion.

Different splanchnic organs may have heterogeneous microcirculatory responses to fluid therapy. For instance, we recently observed that the intestinal microcirculation was more vulnerable to hemorrhaging and had poorer responses to NS resuscitation compared with the liver, kidney, and gracilis muscle [8]. In support of our previous findings, we also observed that during the resuscitation period, the intestinal microcirculation had a more positive response to fluid resuscitation using HTS, GEL, or HES than to that using NS. Our previous and current studies have indicated that the acute microcirculatory response to fluid therapy in susceptible organs such as the intestine is affected not only by volume effects but also by the biochemical composition. Hypertonic fluid and plasma substitutes, especially synthetic colloids, are the most common compositions for correcting hypovolemia in addition to the crystalloid. HTS was proposed to correct microcirculatory dysfunction and inflammatory effects in a hypovolemic state [1] and has been in clinical use for resuscitating hypovolemic and brain injury patients [18]. It was also reported to improve both macrocirculation and microcirculation in comparison with NS and HES during fluid resuscitation in septic shock patients [19]. In previous experimental studies, HTS has improved myocardial blood flow in a pig model of cardiopulmonary resuscitation [20], increased cerebral blood flow in a rat model of cardiac arrest [21], and reduced mesenteric microcirculatory dysfunction in a rat model of strangulated small bowel obstruction [22]. We additionally found that the microcirculatory blood flow in the serosal muscular layer was the most restored in the HTS group (Figs. 2d and 3d), probably because HTS is more effective in increasing superior mesenteric arterial blood flow [23] and in improving intestinal perfusion with selective vasodilation of precapillary arterioles [24] after hemorrhagic shock. Synthetic colloids have been widely used clinically, and their therapeutic effects on microcirculation have gained substantial attention in human studies [1]. GEL was reported to improve splanchnic perfusion in patients who underwent abdominal aortic aneurysm repair [25] and in hypovolemic septic patients [26]. In addition, HES improved gastric mucosal perfusion in patients who received abdominal aortic surgery [27] or liver surgery [28] and improved sublingual microcirculation during early goal-directed therapy for septic patients [29]. However, the effects of synthetic colloids on the microcirculation among multiple splanchnic organs have been less frequently investigated. Our results are in accordance with those of clinical reports indicating that splanchnic microcirculatory blood flow improved after synthetic colloid resuscitation. Synthetic colloids may exert this effect by inducing a decrease in erythrocyte aggregation, thereby reducing the low-shear viscosity of the blood [30]. Despite the microcirculatory advantages of fluid resuscitation using synthetic colloids, concerns remain about the safety of both GEL [13] and HES [11, 12], mainly an increased risk of acute kidney injury, especially in critically ill patients who are vulnerable to oxidative stress. Moreover, we determined that increased reperfusion-induced renal ROS formation may be a mechanism underlying the risk of kidney injury during fluid resuscitation using synthetic colloids.

An ideal resuscitation fluid should not only be effective in restoring both macrocirculation and microcirculation but also cause less reperfusion injury [31]. Recently, Chen and colleague reported that fluid resuscitation with HES 130/0.4 after hemorrhagic shock was associated with lesser oxidative stress and a less severe inflammatory response in the liver, intestine, lungs, and brain compared with GEL and HES 200/0.5 [32]. By contrast, in the current study, increased formation of renal ROS was evident after fluid resuscitation using GEL and HES for hemorrhagic shock. The differences are likely related to two aspects. First, the comparison among resuscitation fluids was not limited to synthetic colloids in the current study; particularly, HTS was included in the comparison. Second, the target organ was different. ROS formation in the kidney was emphasized in the current study because infusion of GEL and, particularly, HES is associated with acute kidney injury [11–13, 33]. ROS have an extremely short lifetime, and there are various antioxidants in vivo. Therefore, a general method for detecting the products of lipid peroxidation, such as malondialdehyde, in tissue may be insufficiently sensitive for detecting acute changes in ROS during reperfusion through fluid resuscitation. In the current study, to evaluate ROS production, we used an enhanced CL method that is highly sensitive for detecting acute changes in ROS [34]. Greater reperfusion-induced ROS formation than that induced by ischemia may be inevitable after effective microcirculatory restoration; accordingly, greater ROS formation was observed in the HTS, GEL, and HES groups than in the control and NS groups. However, the higher reperfusion-induced ROS may not be completely explained by more effective microcirculatory restoration, because HTS was comparably effective in restoring splanchnic microcirculation but did not induce higher renal ROS formation than the synthetic colloids. It may be because of the anti-inflammatory properties of HTS. Studies have reported that using HTS is associated with less neutrophil activation [22] and less expression of genes implicated in leukocyte–endothelium interaction [35]. However, our results should be cautiously applied to clinical scenarios, because there are differences in susceptibility to oxidative challenge between rats and humans [36]. Rodents may be more resistant to the pathological effects of nitrosative stress, but humans may have evolved counter-regulatory mechanisms [37]. Additional investigations may be warranted for understanding the extent of renal ROS formation caused by using synthetic colloids in clinical settings.

In hemorrhagic shock, stabilization of macrocirculatory hemodynamic parameters, such as the MAP, is likely to occur at the expense of splanchnic microcirculatory perfusion. For instance, Dubin and colleagues reported a dissociation between macrocirculation, sublingual and intestinal microcirculation during hemorrhaging; the MAP and arterial pH were significantly modified only at the final stage of bleeding, but microcirculation decreased at the first stage of bleeding [38]. In the current study, we found that the intestine was the most vulnerable splanchnic tissue during the dissociation between macrocirculation and microcirculation by conducting a multiple organ model, which may be the major difference in the current study compared to other experimental models. The simultaneously monitoring of microcirculation among multiple organs was performed using LSCI. The LSCI enables full-field imaging with multiple ROIs and investigating multiple organs in near real time. Furthermore, because a larger ROI can be set, LSCI reduces inter-site and inter-individual variability and can provide comparable or even improved reproducibility of microcirculation compared with other techniques, such as sidestream dark-field imaging [39]. However, sidestream dark-field imaging enables direct visual observation of red blood cells flowing through individual capillaries and can depict heterogeneity between capillaries.

This study has certain limitations. The major limitation is the brief period of observation, because the long laparotomy for exposure of multiple splanchnic organs is associated with significant injury and stress. Therefore it was focused on the period of acute resuscitation; long-term outcomes, such as a survival rate, were less appropriate to evaluate. The improvement of outcomes after fluid resuscitation may be associated with other factors in addition to microcirculatory response and reperfusion injury. For instance, the outcome of resuscitation using HTS was reported to be non-significantly more favorable than that of resuscitation using NS in patients receiving out-of-hospital resuscitation for traumatic hemorrhagic shock [40]. Second, this study compared the microcirculatory responses of various splanchnic organs to different resuscitation fluids. Thus, the euvolemic model was applied. Therefore, the results of the current study may not be generalizable to other methods of treatment such as hypotensive resuscitation, which may improve survival after hemorrhagic shock [31]. Third, the microcirculation plays a crucial role in acute kidney injury [41]. LSCI may enable detection of the heterogeneity in reperfusion dynamics in renal microvascular perfusion [42], but we did not correlate this heterogeneity with renal ROS formation after synthetic colloid resuscitation in the current study. Because the primary goal of the current study was to evaluate the microcirculatory changes among multiple splanchnic organs, heterogeneity in a specific single organ was not examined. However, because the LSCI perfusion distributions of reperfusion correlated to the changes in the mean value of the entire kidney [42] and the renal ROI in the current study was close to that of the entire kidney, the changes in the mean microcirculatory blood flow presented in this study may still correlate to reperfusion-induced microvascular heterogeneity. Fourth, the current study examined two severities of hemorrhagic shock (30 mL/kg and 20 mL/kg). Most investigators attempt to recreate hemorrhagic shock by inducing blood volume loss of more than 40 % [43] because this level of shock is strongly correlated to outcomes. In the current study, this extent of blood loss was reached only in part I of the experiment because volatile anesthesia could not be used during the in vivo ROS measurement. However, it is rational to assume that more reperfusion-induced renal ROS formation occurs when more blood is withdrawn and more fluid is used for resuscitation. Finally, the serum hemoglobin level calculated through arterial blood gas analysis at T_2_ was higher in the GEL group than in the other groups. Although the values obtained using an arterial blood gas analyzer are reliable for use in detecting serial serum lactatemia changes [44], the obtained hemoglobin values should be interpreted with caution and confirmed using standard venous samples [45].

## Conclusions

Our study suggested that even though fluid resuscitation with a crystalloid effectively restored the MAP and decreased serum lactatemia and kidney microcirculation in hemorrhagic shock, the intestinal microcirculation was restored only by other volume expanders, 3 % HTS and synthetic colloids (GEL and HES). In addition, reperfusion-induced formation of renal ROS in vivo was significantly increased in rats resuscitated with the synthetic colloids. Our result supports the clinical literature proposing that fluid resuscitation may increase the risk of kidney injury.

## Key messages

- Hemorrhagic shock induced heterogeneous splanchnic microcirculatory derangement in the kidney and, particularly, the intestine; by contrast, the liver was less affected
- Fluid resuscitation with 0.9 % saline, 3 % saline, and synthetic colloids effectively restored microcirculation in the kidney, blood pressure and serum lactate levels in hemorrhagic shock
- The intestinal microcirculation was restored using 3 % saline and synthetic colloids, but not 0.9 % saline
- Fluid resuscitation using the synthetic colloids was associated with significantly increased reperfusion-induced formation of renal reactive oxygen species in vivo

## Additional file

Additional file 1:
**The protocol of setting regions of interest and its' intra- and inter-observer's agreement.** (DOCX 357 kb)

## Abbreviations

ANOVA: analysis of variance
CL: chemiluminescence
GEL: gelatin
HES: hydroxyethyl starch
HR: heart rate
HTS: hypertonic saline
LSCI: laser speckle contrast imaging
MAP: mean arterial pressure
MCLA: 2-methyl-6-[4-methoxyphenyl]-3,7-dihydroimidazo-[1,2-a]-pyrazin-3-one hydrochloride
NS: 0.9 % saline
PaCO_2_: arterial partial pressure of carbon dioxide
PaO_2_/FiO_2_: ratio of arterial partial pressure of oxygen/inspired oxygen fraction
PU: perfusion unit
ROI: region of interest
ROS: reactive oxygen species
T_0_: time zero
T_1_: 15–60 minutes after time zero
T_2_: 2 h after fluid resuscitation
