# Supplementary material for: Effects of different types of fluid resuscitation for hemorrhagic shock on splanchnic organ microcirculation and renal reactive oxygen species formation
Source: Crit Care. 2015 Dec 11;19:434. doi: 10.1186/s13054-015-1135-y (PMC4699328; doi:10.1186/s13054-015-1135-y)
Supplement: Additional file 1: — The protocol of setting regions of interest and its' intra- and inter-observer's agreement. (DOCX 357 kb) [file 13054_2015_1135_MOESM1_ESM.docx]

1. Anatomic landmarks for determining regions of interest (ROIs)
2. The left liver was identified according to the hepatic fissure and falciform ligament. The hepatic ROI was set 1.5 cm to the left of the hepatic fissure.
3. The entire left kidney was exposed and identified through direct visualization; the ROI was set to cover both the short and long axes of the exposed kidney.
4. The selected terminal ileum was approximately 6 to 10 cm proximal to the ileocecal valve of each rat. The section was 2 cm in length and defined on the antimesenteric aspect of the intestinal lumen.
5. The ROIs did not cover the outer margin of the intestine because the multiple-organ model in the current study involved stretching forces for fully exposing the terminal ileum. Because mesenteric blood flow has a gray zone that causes the distal portion of supplied tissue to be susceptible to a stretch-induced decrease of blood flow, ROIs were selected on the central portion of exposed tissue, and we carefully avoided including the outmost margin of each tissue in the ROIs. In addition, using a high-frequency desiccator to expose the intestine enabled confining the surgical trauma to a small and restricted outer margin.
6. The same intestinal ROIs were selected for each of the rats as follows: the ROI of the mucosa was set to cover as much of the mucosa as possible; the ROI of the intestinal serosal muscular layer was at the midline of the antimesenteric aspect (1 to 2 cm from the mucosa); and the ROI of the Peyer’s patch was identified by visualizing the clusters of lymph nodes.
7. The skin (2-cm incision) removed to expose the gracilis muscle was approximately 3 cm lateral to the midline of the abdomen (the midline was identified according to the connection between the xiphoid bone and anus).
8. In this study, each ROI was chosen by the first author and one technician who had more than 3 years of experience in using laser speckle contrast imaging in a rat model. The MoorFLPI full-field laser perfusion imager provides nearly real-time images, which enable investigators to relocate the monitoring ROIs (illustrated in the following figure) in real time. During measurement, we reviewed the ROIs every 10 min and relocated them according to the aforementioned rules. Regularly reviewing ROIs may facilitate minimizing the bias caused by sampling variations.


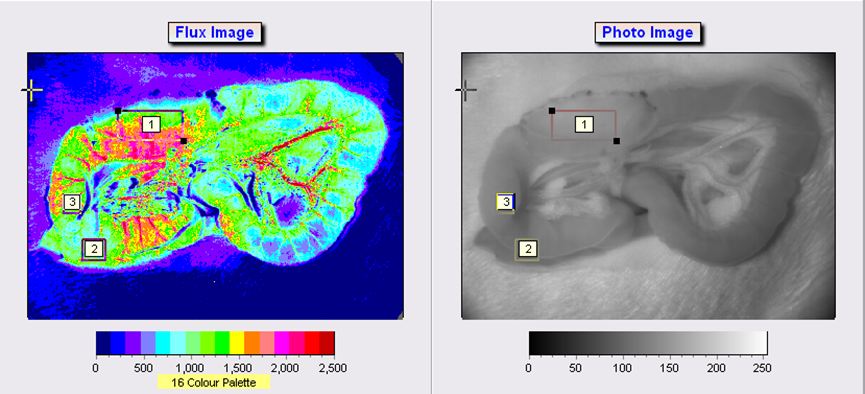


1. Reproducibility and repeatability

The reproducibility and repeatability of microcirculatory blood flow values in the six ROIs were calculated by the second author according to the previously described protocol for determining ROIs.

| Region of interest | ICC^*^ (95% CI) | Weighted kappa (95% CI)^#^ | Within subject CV (%)^^^ |
| --- | --- | --- | --- |
| 1. Liver | 0.902 (0.8019 to 0.9591) | 0.769 (0.628 to 0.910) | 5.04 |
| 2. Kidney | 0.940 (0.8750 to 0.9753) | 0.769 (0.648 to 0.891) | 6.84 |
| 3. Mucosa | 0.987 (0.9722 to 0.9948) | 0.882 (0.849 to 0.916) | 8.67 |
| 4. Serosal muscular layer | 0.965 (0.9249 to 0.9856) | 0.817 (0.738 to 0.896) | 9.13 |
| 5. Peyer's patch | 0.953 (0.8999 to 0.9805) | 0.805 (0.712 to 0.899) | 11.10 |
| 6. Gracilis muscle | 0.836 (0.6825 to 0.9294) | 0.662 (0.523 to 0.800) | 15.00 |

*The ICC (intraclass correlation coefficient) represents the degree of consistency among measurements, and > 0.75 represents excellent agreement.

# The value of weighted kappa represents the interobserver agreement. Kappa = 1 represents perfect agreement; kappa= 0.81–0.99 represents excellent agreement; kappa = 0.61–0.80 represents good agreement, and kappa = 0.41–0.60 represents moderate agreement.^ CV = coefficient of variation. CVs < 35% are acceptable.
